# Jahn–Teller coupling to moiré phonons in the continuum model formalism for small-angle twisted bilayer graphene

**DOI:** 10.1140/epjp/s13360-020-00647-7

**Published:** 2020-08-06

**Authors:** Mattia Angeli, Michele Fabrizio

**Affiliations:** International School for Advanced Studies (SISSA), Via Bonomea 265, 34136 Trieste, Italy

## Abstract

We show how to include the Jahn–Teller coupling of moiré phonons to the electrons in the continuum model formalism which describes small-angle twisted bilayer graphene. These phonons, which strongly couple to the valley degree of freedom, are able to open gaps at most integer fillings of the four flat bands around the charge neutrality point. Moreover, we derive the full quantum mechanical expression of the electron–phonon Hamiltonian, which may allow accessing phenomena such as the phonon-mediated superconductivity and the dynamical Jahn–Teller effect.

## Introduction

The discovery of superconductivity first in small-angle twisted bilayer graphene (tBLG) [1–4], and later in trilayer [5] and double bilayer graphene [6], has stimulated an intense theoretical and experimental research activity. In these systems, the twist angle tunes a peculiar interference within a large set of energy bands, compressing energy levels to form a set of extremely narrow bands around charge neutrality [7–10]. In twisted bilayer graphene, these flat bands (FBs) have a bandwidth of the order of $\approx 10-20$ meV and are isolated in energy by single-particle band gaps of the order of 30–50 meV. Superconductivity is observed upon doping such narrow bands, often surrounding insulating states at fractional fillings that contradict the metallic behaviour predicted by band structure calculations. The observed phenomenology of these insulating states, which turn metallic above a threshold Zeeman splitting or above a critical temperature, suggests that they might arise from a weak-coupling Stoner or CDW band instability driven by electron–electron and/or electron–phonon interactions, rather than from the Mott’s localisation phenomenon in the presence of strong correlations. This is further supported by noting that the effective *on-site* Coulomb repulsion *U* must be identified with the charging energy of the supercell, which can be as large as tens of nanometers at small angles, projected onto the flat bands. If screening effects due to the gates and to the other bands are taken into account, the actual value of *U* is estimated of the order of few meVs, suggesting that tBLG might not be more correlated than a single graphene sheet [11]. On the contrary, there are evidences that the coupling to the lattice is instead anomalously large if compared with the FBs bandwidth. For instance, ab initio DFT-based calculations fail to predict well-defined FBs separated from other bands, unless atomic positions are allowed to relax [7, 9, 12–15], in which case gaps open that are larger than the FBs bandwidth. Further evidences supporting a sizeable electron–phonon coupling come from transport properties [16–18], but also from direct electronic structure calculations.

Specifically, in Ref. [8] it was shown that a pair of optical phonon modes are rather strongly coupled to the FBs and thus might play an important role in the physics of tBLG. These modes, which have a long wavelength modulation on the same moiré scale, have been dubbed as “moiré phonons” and recently observed experimentally [19]. However, the large number of atoms contained in the small-angle unit cell of twisted bilayer graphene (more than 11000) makes any calculation more involved than a simple tight-binding one, rather tough, if not computationally impossible. In this paper, we try to cope with such problem by implementing the effect of these phonon modes on the band structure in the less computationally demanding continuum model of Ref. [20]. This method can serve as a suitable starting point for BCS [21, 22], Hartree-Fock [23–26] and many other calculations, which may involve both phonons and correlations. The work is organised as follows. In Sect. 2, we derive the Bistritzer and MacDonald continuum model for twisted bilayer graphene. In Sect. 3, we implement in the continuum model formalism the effect of a static atomic displacement. By using lattice deformation fields which are similar to the Jahn–Teller moiré phonon modes of Ref. [8], we show how the band structure and the density of states of the system evolve as a function of the lattice distortion intensity. Finally, 4 is devoted to concluding remarks.

## Continuum model Hamiltonian

We start by introducing the Bistritzer–MacDonald continuum model for twisted bilayer graphene [20] and recall that the single-layer Dirac Hamiltonian is, around the ${\mathbf {K}}$ and ${\mathbf {K}}'=-{\mathbf {K}}$ valleys, respectively,

1 $$\begin{aligned} \begin{aligned} {\hat{H}}_{{\mathbf {k}}\sim {\mathbf {K}}}&\equiv {\hat{H}}^{+1}_{\mathbf {k}}= -v\,\Big ({\mathbf {k}}-{\mathbf {K}}\Big )\cdot \Big (\sigma _x,\sigma _y\Big )\,, \\ {\hat{H}}_{{\mathbf {k}}\sim -{\mathbf {K}}}&\equiv {\hat{H}}^{-1}_{\mathbf {k}}= -v\,\Big ({\mathbf {k}}+{\mathbf {K}}\Big )\cdot \Big (-\sigma _x,\sigma _y\Big )\,, \end{aligned} \end{aligned}$$

namely

2 $$\begin{aligned} {\hat{H}}^\zeta _{\mathbf {k}}&= -v\,\Big ({\mathbf {k}}-\zeta {\mathbf {K}}\Big )\cdot \Big (\zeta \,\sigma _x,\sigma _y\Big )\,, \end{aligned}$$

with $\zeta =\pm 1$ the valley index, and where the Pauli matrices $\sigma _a$ act on the two component wavefunctions, each component referring to one of the two sites per unit cell that form the honeycomb lattice, which we shall hereafter denote as sublattices *A* and *B*.

**Fig. 1:**
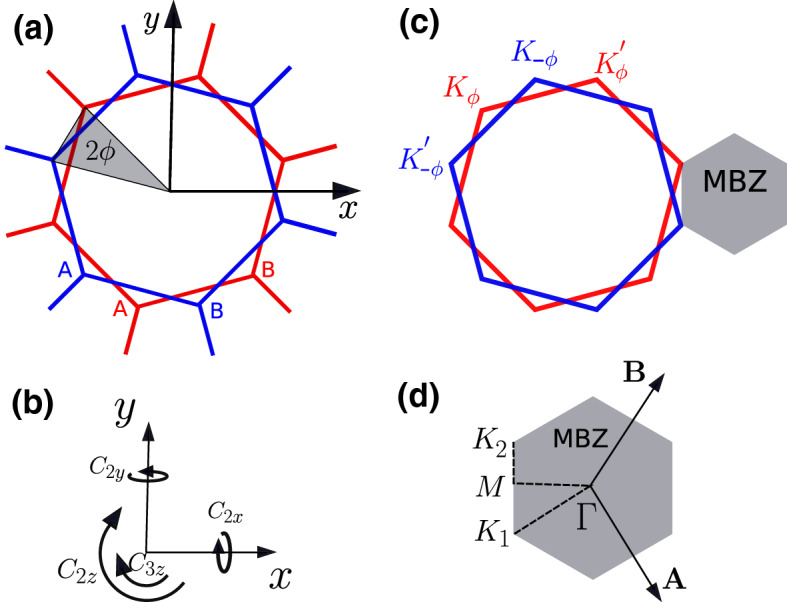
**a** Two graphene layers twisted with respect to the centre of two overlapping hexagons by an angle $2\phi$. **b** Symmetries of twisted bilayer graphene which are those of the group $\text {D}_6$. Two- ($180^\circ$) and threefold ($120^\circ$) rotations with respect to the *z*-axis denoted as $C_{2z}$ and $C_{3z}$ as well as twofold rotations with respect to the *x*- and *y*-axis denoted as $C_{2y}$ and $C_{2x}$. **c** The monolayer Brillouin zones that are folded in the mini-Brillouin zone (MBZ) of the twisted heterostructure. **d** Mini-Brillouin zone of twisted bilayer graphene with reciprocal lattice vectors ${\mathbf {A}}$ and ${\mathbf {B}}$. The high-symmetry points and paths are highlighted

Our analysis must start by defining the specific twisted bilayer graphene we shall investigate, and by setting some conventional notations. We assume that the twisted bilayer is obtained by rotating two AA-stacked layers ($i=1,2$) by an opposite angle $\pm \phi$ with respect to the centre of two overlapping hexagons, see Fig. 1a, where $\tan \phi = 1/\big [(2k+1)\sqrt{3}\big ]$, with large positive integer *k*. With this choice, the moiré pattern forms a superlattice, which is still honeycomb and endowed by a $\text {D}_6$ space group symmetry [7] that is generated by the threefold rotation $\text {C}_{3z}$ around the *z*-axis perpendicular to the bilayer and the twofold rotations around the in-plane *x*- and *y*-axes, $\text {C}_{2x}$ and $\text {C}_{2y}$, respectively.

The corresponding mini-Brillouin zone (MBZ) has reciprocal lattice vectors

3 $$\begin{aligned} {\mathbf {A}}={\mathbf {G}}_2^{(2)}-{\mathbf {G}}_2^{(1)}\,, \quad {\mathbf {B}}={\mathbf {G}}_1^{(1)}-{\mathbf {G}}_1^{(2)}\,. \end{aligned}$$

where ${\varvec{G}}^{(1)}_{1/2}=\mathscr {R}_{+\phi }\big ({\varvec{G}}_{1/2}\big )$ and ${\varvec{G}}^{(2)}_{1/2}=\mathscr {R}_{-\phi }\big ({\varvec{G}}_{1/2}\big )$ are the reciprocal lattice vectors of each layer after the twist, with $\mathscr {R}_\phi$ the rotation operator by an angle $\phi$.

The Dirac nodes of each monolayer are, correspondingly, $\mathscr {R}_{\pm \phi }({\mathbf {K}})\equiv {\mathbf {K}}_{\pm \phi }$ for the valley we shall denote as $\zeta =+1$, and $\mathscr {R}_{\pm \phi }({\mathbf {K}}')\equiv {\mathbf {K}}'_{\pm \phi }$ for the other valley, $\zeta =-1$. With our choices, ${\mathbf {K}}_{+\phi }$ and ${\mathbf {K}}'_{-\phi }$ fold into the same point ${\mathbf {K}}_2$ of the MBZ, as well as ${\mathbf {K}}_{-\phi }$ and ${\mathbf {K}}'_{+\phi }$ into the point ${\mathbf {K}}_1$, see Fig. 1.

We introduce the (real) Wannier functions derived by the $p_z$ orbital of each carbon atom:

4 $$\begin{aligned} \begin{aligned} \phi _{1\, \alpha {\mathbf {R}}^{(1)}}({\mathbf {r}})&\equiv \phi \bigg ({\mathbf {r}}-{\mathbf {R}}^{(1)}-{\mathbf {r}}^{(1)}_\alpha -\frac{\displaystyle {\varvec{d}}_\perp }{\displaystyle 2}\bigg )\,, \\ \phi _{2\,\alpha {\mathbf {R}}^{(2)}}({\mathbf {r}})&\equiv \phi \bigg ({\mathbf {r}}-{\mathbf {R}}^{(2)}-{\mathbf {r}}^{(2)}_\alpha +\frac{\displaystyle {\varvec{d}}_\perp }{\displaystyle 2}\bigg )\,, \end{aligned} \end{aligned}$$

where ${\varvec{d}}_\perp = (0,0,d)$, with *d* the interlayer distance, ${\mathbf {R}}^{(i)}$ label the positions of the unit cells in layer $i=1,2$, while ${\mathbf {r}}^{(i)}_\alpha$ the coordinates with respect to ${\mathbf {R}}^{(i)}$ of the two sites within each unit cell, $\alpha =A,B$ denoting the two sublattices. From the Wannier functions, we build the Bloch functions

5 $$\begin{aligned} \begin{aligned} \psi _{1\,\alpha {\mathbf {k}}}({\mathbf {r}})&= \frac{\displaystyle 1}{\displaystyle \sqrt{N}}\,\sum _{{\mathbf {R}}^{(1)}}\, \text {e}^{-i{\mathbf {k}}\cdot \big ({\mathbf {R}}^{(1)}+{\mathbf {r}}^{(1)}_\alpha \big )}\; \phi _{1\, \alpha {\mathbf {R}}^{(1)}}({\mathbf {r}})\,, \\ \psi _{2\,\alpha {\mathbf {k}}}({\mathbf {r}})&= \frac{\displaystyle 1}{\displaystyle \sqrt{N}}\,\sum _{{\mathbf {R}}^{(2)}}\, \text {e}^{-i{\mathbf {k}}\cdot \big ({\mathbf {R}}^{(2)}+{\mathbf {r}}^{(2)}_\alpha \big )}\; \phi _{2\,\alpha {\mathbf {R}}^{(2)}}({\mathbf {r}})\,. \end{aligned} \end{aligned}$$

Conventionally, one assumes the two-centre approximation [20], so that, if $V_\perp ({\mathbf {r}})$ is the interlayer potential, then the interlayer hopping

6 $$\begin{aligned} \int \mathrm{{d}}{\mathbf {r}}\, \phi _{1\, \alpha {\mathbf {R}}^{(2)}}({\mathbf {r}})\;V_\perp ({\mathbf {r}})\; \phi _{2\,\beta {\mathbf {R}}^{(1)}}({\mathbf {r}})&\simeq T_\perp \Big ({\mathbf {R}}^{(2)}+{\mathbf {r}}^{(2)}_\alpha - {\mathbf {R}}^{(1)}-{\mathbf {r}}^{(1)}_\beta \Big )\,, \end{aligned}$$

depends only on the distance between the centres of the two Wannier orbitals. We define $T_\perp ({\mathbf {q}})$, the Fourier transform of $T_\perp ({\mathbf {r}})$:

7 $$\begin{aligned} T_\perp ({\mathbf {r}}) = \frac{\displaystyle 1}{\displaystyle N}\,\sum _{\mathbf {q}}\, \text {e}^{i{\mathbf {q}}\cdot {\mathbf {r}}}\;T_\perp ({\mathbf {q}})\,, \end{aligned}$$

where ${\mathbf {r}}$ and ${\mathbf {q}}$ are vectors in the *x*–*y* plane. Hereafter, all momenta are assumed also to lie in the *x*–*y* plane.

The interlayer hopping between an electron in layer 1 with momentum ${\mathbf {p}}$ and one in layer 2 with momentum ${\mathbf {k}}$ is in general a matrix ${\hat{T}}_{{\mathbf {k}}{\mathbf {p}}}$, with elements $T^{\alpha \beta }_{{\mathbf {k}}{\mathbf {p}}}$, $\alpha ,\beta =A,B$, which, through equations (5), (6) and (7), reads explicitly

8 $$\begin{aligned} T^{\alpha \beta }_{{\mathbf {k}}{\mathbf {p}}}&= \frac{\displaystyle 1}{\displaystyle N}\,\sum _{{\mathbf {R}}^{(2)}{\mathbf {R}}^{(1)}}\, \text {e}^{-i{\mathbf {k}}\cdot \big ({\mathbf {R}}^{(2)}+{\mathbf {r}}^{(2)}_\alpha \big )}\; \text {e}^{i{\mathbf {p}}\cdot \big ({\mathbf {R}}^{(1)}+{\mathbf {r}}^{(1)}_\beta \big )}\; T_\perp \big ({\mathbf {R}}^{(2)}+{\mathbf {r}}^{(2)}_\alpha - {\mathbf {R}}^{(1)}-{\mathbf {r}}^{(1)}_\beta \big ) \nonumber \\&= \sum _{\mathbf {q}}\, T_\perp (-{\mathbf {q}})\;\; \frac{\displaystyle 1}{\displaystyle N^2}\,\sum _{{\mathbf {R}}^{(2)}{\mathbf {R}}^{(1)}}\, \text {e}^{-i\big ({\mathbf {k}}+{\mathbf {q}}\big )\cdot \big ({\mathbf {R}}^{(2)}+{\mathbf {r}}^{(2)}_\alpha \big )}\;\text {e}^{i\big ({\mathbf {p}}+{\mathbf {q}}\big ) \cdot \big ({\mathbf {R}}^{(1)}+{\mathbf {r}}^{(1)}_\beta \big )} \nonumber \\&= \sum _{\mathbf {q}}\, T_\perp (-{\mathbf {q}})\;\sum _{{\mathbf {G}}^{(2)}{\mathbf {G}}^{(1)}}\, \delta _{{\mathbf {k}}+{\mathbf {q}}\,,\,-{\mathbf {G}}^{(2)}}\,\delta _{{\mathbf {p}}+{\mathbf {q}}\,,\,-{\mathbf {G}}^{(1)}}\, \text {e}^{i{\mathbf {G}}^{(2)}\cdot {\mathbf {r}}^{(2)}_\alpha }\;\text {e}^{-i{\mathbf {G}}^{(1)}\cdot {\mathbf {r}}^{(1)}_\beta } \nonumber \\&= \sum _{{\mathbf {G}}^{(2)}{\mathbf {G}}^{(1)}}\,T_\perp \Big ({\mathbf {k}}+{\mathbf {G}}^{(2)}\Big )\; \delta _{{\mathbf {k}}+{\mathbf {G}}^{(2)}\,,\,{\mathbf {p}}+{\mathbf {G}}^{(1)}}\;\text {e}^{i{\mathbf {G}}^{(2)}\cdot {\mathbf {r}}^{(2)}_\alpha }\; \text {e}^{-i{\mathbf {G}}^{(1)}\cdot {\mathbf {r}}^{(1)}_\beta }\;. \end{aligned}$$

Since we are interested in the low energy physics, ${\mathbf {k}}$ and ${\mathbf {p}}$ must be close to the corresponding Dirac points, namely ${\mathbf {K}}_\phi$ and ${\mathbf {K}}'_\phi$ for ${\mathbf {p}}$ in layer 1, while ${\mathbf {K}}_{-\phi }$ and ${\mathbf {K}}'_{-\phi }$ for ${\mathbf {k}}$ in layer 2. Therefore, ${\hat{T}}_{{\mathbf {k}}{\mathbf {p}}}$ can in principle couple to each other states of different layers within the same valley or between opposite valleys. Since $T_\perp ({\mathbf {q}})$ decays exponentially with $q=|{\mathbf {q}}|$ [20], the leading terms are those with the least possible $\big |{\mathbf {k}}+{\mathbf {G}}^{(2)}\big |$ compatible with momentum conservation ${\mathbf {k}}+{\mathbf {G}}^{(2)}={\mathbf {p}}+{\mathbf {G}}^{(1)}$. At small twist angle $\phi$, only the intra-valley matrix elements, ${\mathbf {p}}\sim {\mathbf {k}}$, are sizeable, while the inter-valley ones are negligibly small, despite opposite valleys of different layers fold into the same point of the MBZ. For instance, if ${\mathbf {p}}\simeq {\mathbf {K}}_{+\phi }$ and ${\mathbf {k}}\simeq {\mathbf {K}}'_{-\phi }$, momentum conservation requires very large ${\mathbf {G}}^{(1)}= (2k+1)\,\big ({\mathbf {G}}_2^{(1)}-{\mathbf {G}}_1^{(1)}\big )$ and ${\mathbf {G}}^{(2)}=(2k+1)\,\big ({\mathbf {G}}_2^{(2)}-{\mathbf {G}}_1^{(2)}\big )$, thus an exponentially small $T_\perp \big ({\mathbf {k}}+{\mathbf {G}}^{(2)}\big )$. The effective decoupling between the two valleys implies that the number of electrons within each valley is to high accuracy a conserved quantity, thus an emergent valley $U_v(1)$ symmetry [20, 33] that causes accidental band degeneracies along high-symmetry lines in the MBZ [8, 33].

We can therefore just consider the intra-valley inter-layer scattering processes. We start with valley $\zeta =+1$ and thus require that ${\mathbf {k}}$ is close to ${\mathbf {K}}_{-\phi }={\mathbf {K}}_1$ and ${\mathbf {p}}$ close to ${\mathbf {K}}_{+\phi }={\mathbf {K}}_2$, see Fig. 1d. Since the modulus of ${\mathbf {k}}\sim {\mathbf {K}}_1$ is invariant under $C_{3z}$ rotations, where $C_{3z}\big ({\mathbf {K}}_1\big ) = {\mathbf {K}}_1-{\mathbf {G}}^{(2)}_1$ and $C_{3z}^2\big ({\mathbf {K}}_1\big ) = {\mathbf {K}}_1-{\mathbf {G}}^{(2)}_2$, maximisation of $T_\perp \big ({\mathbf {k}}+{\mathbf {G}}^{(2)}\big )$ compatibly with momentum conservation leads to the following conditions, see Eq. (3),

9 $$\begin{aligned} \begin{aligned} {\mathbf {p}}&= {\mathbf {k}}\,, \\ {\mathbf {p}}&= {\mathbf {k}}- {\mathbf {G}}^{(2)}_1 + {\mathbf {G}}^{(1)}_1 ={\mathbf {k}}+{\varvec{B}}\,, \\ {\mathbf {p}}&= {\mathbf {k}}- {\mathbf {G}}^{(2)}_2 + {\mathbf {G}}^{(1)}_2 = {\mathbf {k}}- {\varvec{A}}\,. \end{aligned} \end{aligned}$$

Upon defining $T({\mathbf {k}})\equiv t_\perp$, and using Eq. (9) to evaluate the phase factors in (8), we finally obtain

10 $$\begin{aligned} {\hat{T}}_{{\mathbf {k}}{\mathbf {p}}}^{\zeta =+1} = \delta _{{\mathbf {p}},{\mathbf {k}}}\;{\hat{T}}_1 + \delta _{{\mathbf {p}}\,,\,{\mathbf {k}}+{\varvec{B}}}\; {\hat{T}}_2 + \delta _{{\mathbf {p}}\,,\,{\mathbf {k}}-{\varvec{A}}}\;{\hat{T}}_3\;, \end{aligned}$$

where we explicitly indicate the valley index $\zeta$, and

11 $$\begin{aligned} {\hat{T}}_1 = t_\perp \, \begin{pmatrix} 1 &{} 1\\ 1 &{} 1 \end{pmatrix}\;, \quad {\hat{T}}_2 =t_\perp \,\begin{pmatrix} 1&{} \omega ^* \\ \omega &{}1 \end{pmatrix}\,, \quad {\hat{T}}_3 = t_\perp \,\begin{pmatrix} 1 &{} \omega \\ \omega ^* &{} 1 \end{pmatrix}\,, \end{aligned}$$

with $$\omega =\text {e}^{2\pi i/3}$$.

We now focus on the other valley, $\zeta =-1$, and take ${\mathbf {k}}$ close to ${\mathbf {K}}'_{-\phi }=-{\mathbf {K}}_2$, and ${\mathbf {p}}$ to ${\mathbf {K}}'_{\phi }=-{\mathbf {K}}_1$, see Fig. 1d. In this case, Eq. (9) is replaced by

12 $$\begin{aligned} {\mathbf {p}}= {\mathbf {k}}\,, \quad {\mathbf {p}}= {\mathbf {k}}-{\varvec{B}}\,, \quad {\mathbf {p}}= {\mathbf {k}}+ {\varvec{A}}\,, \end{aligned}$$

and

13 $$\begin{aligned} {\hat{T}}_{{\mathbf {k}}{\mathbf {p}}}^{\zeta =-1} = \delta _{{\mathbf {p}},{\mathbf {k}}}\;{\hat{T}}_1^* + \delta _{{\mathbf {p}}\,,\,{\mathbf {k}}-{\varvec{B}}}\; {\hat{T}}_2^* + \delta _{{\mathbf {p}}\,,\,{\mathbf {k}}+{\varvec{A}}}\;{\hat{T}}_3^*\;. \end{aligned}$$

Let us briefly discuss how one can take into account lattice relaxation, which is known to shrink the energetically unfavourable AA regions enlarging the Bernal-stacked triangular domains in the moiré pattern [7, 12, 14, 15, 27, 28, 30, 31]. As a consequence, the inter- and intra-sublattice hopping processes acquire different amplitudes, which is taken into account by modifying the operators ${\hat{T}}_i$ in Eq. (11) according to

14 $$\begin{aligned} \begin{aligned} {\hat{T}}_1&\rightarrow T_1(u,u')= \begin{pmatrix} u &{} u'\\ u' &{} u \end{pmatrix} = u\,\sigma _0 + u'\,\sigma _x\;, \\ {\hat{T}}_2&\rightarrow {\hat{T}}_2(u,u')=\begin{pmatrix} u&{} u'\,\omega ^* \\ u'\,\omega &{}u \end{pmatrix}= u\,\sigma _0 + u'\Big ( \cos \frac{\displaystyle 2\pi }{\displaystyle 3}\,\sigma _x + \sin \frac{\displaystyle 2\pi }{\displaystyle 3}\,\sigma _y\Big ) \,, \\ {\hat{T}}_3&\rightarrow {\hat{T}}_3(u,u')= \begin{pmatrix} u &{} u'\,\omega \\ u'\,\omega ^* &{} u \end{pmatrix}= u\,\sigma _0 + u'\Big ( \cos \frac{\displaystyle 2\pi }{\displaystyle 3}\,\sigma _x -\sin \frac{\displaystyle 2\pi }{\displaystyle 3}\,\sigma _y\Big )\,, \end{aligned} \end{aligned}$$

with *u* generally smaller than $u'$.

We conclude by showing how this formalism allows recovering the untwisted case, where ${\mathbf {G}}^{(1)}_{1/2}={\mathbf {G}}^{(2)}_{1/2}$, so that, through Eq. (3), ${\varvec{A}}={\varvec{B}}={\varvec{0}}$, and therefore

15 $$\begin{aligned} {\hat{T}}_{{\mathbf {k}}{\mathbf {p}}} \;\underset{\phi \rightarrow 0}{\longrightarrow {\,}}\; \delta _{{\mathbf {p}},{\mathbf {k}}}\;\Big ({\hat{T}}_1 +\hat{T}_2+{\hat{T}}_3\Big ) = 3t_\perp \,\delta _{{\mathbf {p}},{\mathbf {k}}}\, \begin{pmatrix} 1 &{} 0\\ 0 &{} 1 \end{pmatrix}\,, \end{aligned}$$

which is what one would expect from an AA-stacked bilayer.

### A more convenient representation

For our purposes, it is actually more convenient to use the alternative representation of the Hamiltonian derived in Ref. [32]. We translate ${\mathbf {K}}'_{\phi }=-{\mathbf {K}}_1$ so that it falls on ${\mathbf {K}}_{-\phi }={\mathbf {K}}_1$, and similarly, ${\mathbf {K}}'_{-\phi }=-{\mathbf {K}}_2$ on ${\mathbf {K}}_{\phi }={\mathbf {K}}_2$, see Fig. 1d. This implies that the diagonal parts of the Hamiltonian ${\hat{H}}^{(i)}_\zeta ({\mathbf {k}})$, where $i=1,2$ is the layer index and $\zeta =\pm 1$ the valley one, become simply

16 $$\begin{aligned} \begin{aligned} {\hat{H}}^{(2)}_{+1}({\mathbf {k}})&= - v\, \Big ({\mathbf {k}}-{\mathbf {K}}_1\Big )\cdot \Big (\sigma _x\,,\,\sigma _y\Big )\,, \\ {\hat{H}}^{(1)}_{-1}({\mathbf {k}})&= - v\, \Big ({\mathbf {k}}-{\mathbf {K}}_1\Big )\cdot \Big (-\sigma _x\,,\,\sigma _y\Big ) = v\, \Big ({\mathbf {k}}-{\mathbf {K}}_1\Big )\cdot \varvec{\sigma }^T\,, \\ {\hat{H}}^{(1)}_{+1}({\mathbf {k}})&= - v\, \Big ({\mathbf {k}}-{\mathbf {K}}_2\Big )\cdot \Big (\sigma _x\,,\,\sigma _y\Big )\,, \\ {\hat{H}}^{(2)}_{-1}({\mathbf {k}})&= - v\, \Big ({\mathbf {k}}-{\mathbf {K}}_2\Big )\cdot \Big (-\sigma _x\,,\,\sigma _y\Big ) =v\, \Big ({\mathbf {k}}-{\mathbf {K}}_2\Big )\cdot \varvec{\sigma }^T \,. \end{aligned} \end{aligned}$$

**Fig. 2:**
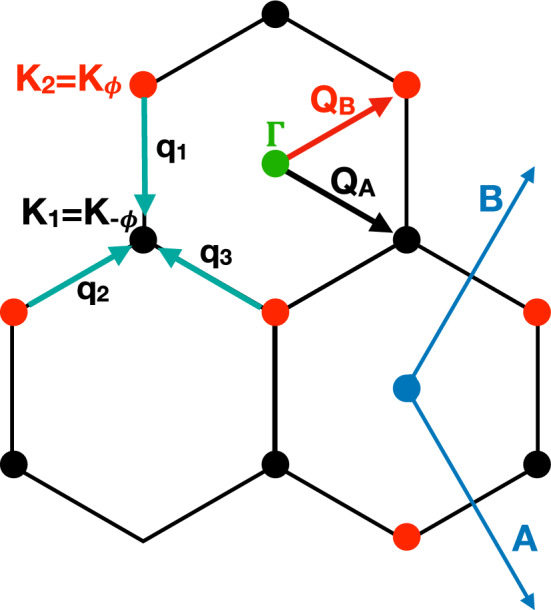
The lattice in momentum space of Ref. [32]. The ${\mathbf {Q}}_{A}$ and ${\mathbf {Q}}_{B}$ vectors span the lattice formed by the ${\mathbf {K}}$ points of the two twisted monolayer Brillouin zones

Following Ref. [32], we define a set of vectors

17 $$\begin{aligned} {\mathbf {Q}}&= \Big \{{\mathbf {Q}}_A\,,\,{\mathbf {Q}}_B\Big \}= {\left\{ \begin{array}{ll} {\mathbf {Q}}_A = {\mathbf {K}}_1 + n{\mathbf {A}}+ m{\mathbf {B}}\,,\\ {\mathbf {Q}}_B = {\mathbf {K}}_2 + n{\mathbf {A}}+ m{\mathbf {B}}\,, \end{array}\right. } \end{aligned}$$

which span the vertices of the MBZs, where ${\mathbf {Q}}_A$, black circles in Fig. 2, correspond to valley $\zeta =+1$ in layer 2 and valley $\zeta =-1$ in layer 1, while ${\mathbf {Q}}_B$, red circles in Fig. 2, correspond to valley $\zeta =+1$ in layer 1 and valley $\zeta =-1$ in layer 2. In addition, we define

18 $$\begin{aligned} {\mathbf {q}}_1 = {\mathbf {K}}_{1}-{\mathbf {K}}_{2} \quad {\mathbf {q}}_2 = {\mathbf {q}}_1+{\mathbf {B}}\quad {\mathbf {q}}_3 = {\mathbf {q}}_1-{\mathbf {A}}\,. \end{aligned}$$

Next, we redefine the momenta for layers 1 and 2 as, respectively,

19 $$\begin{aligned} {\mathbf {p}}-{\mathbf {K}}_{2} \rightarrow {\mathbf {k}}' - {\mathbf {Q}}_B \quad {\mathbf {k}}-{\mathbf {K}}_{1}\rightarrow {\mathbf {k}}' - {\mathbf {Q}}_A\,, \end{aligned}$$

thus

20 $$\begin{aligned} {\mathbf {k}}= {\mathbf {k}}'+{\mathbf {K}}_{1}-{\mathbf {Q}}_A \quad {\mathbf {p}}= {\mathbf {k}}'+{\mathbf {K}}_{2}-{\mathbf {Q}}_B\,, \end{aligned}$$

so that the selection rules transform into

21 $$\begin{aligned} \begin{aligned} {\mathbf {p}}&= {\mathbf {k}}\;\Rightarrow \; {\mathbf {Q}}_A-{\mathbf {Q}}_B = {\mathbf {q}}_1 \;\Rightarrow \; {\mathbf {Q}}_B = {\mathbf {Q}}_A -{\mathbf {q}}_1 \,, \\ {\mathbf {p}}&= {\mathbf {k}}+{\mathbf {B}}\;\Rightarrow \; {\mathbf {Q}}_A-{\mathbf {Q}}_B = {\mathbf {q}}_1+{\mathbf {B}}\;\Rightarrow \; {\mathbf {Q}}_B = {\mathbf {Q}}_A -{\mathbf {q}}_2\,, \\ {\mathbf {p}}&= {\mathbf {k}}-{\mathbf {A}}\;\Rightarrow \; {\mathbf {Q}}_A-{\mathbf {Q}}_B = {\mathbf {q}}_1-{\mathbf {A}}\;\Rightarrow \; {\mathbf {Q}}_B = {\mathbf {Q}}_A -{\mathbf {q}}_3\,. \end{aligned} \end{aligned}$$

With those definitions, and denoting the conserved momentum ${\mathbf {k}}'$ as ${\mathbf {k}}$, the Hamiltonian of valley $\zeta$ now reads

22 $$\begin{aligned} \hat{H}^\zeta _{{\mathbf {Q}}{\mathbf {Q}}'}({\mathbf {k}})&= \delta _{{\mathbf {Q}}{\mathbf {Q}}'}\,v\,\zeta \,\big ({\mathbf {k}}-{\mathbf {Q}}\big ) \cdot \big (\sigma _x,\zeta \,\sigma _y\big ) \nonumber \\&\quad + \sum _{i=1}^3\,\Big (\delta _{{\mathbf {Q}}'-{\mathbf {Q}},{\mathbf {q}}_i} + \delta _{{\mathbf {Q}}-{\mathbf {Q}}',{\mathbf {q}}_i}\Big )\,{\hat{T}}^\zeta _i(u,u')\,, \end{aligned}$$

where

23 $$\begin{aligned} {\hat{T}}^\zeta _1(u,u')&= u\,\sigma _0 + u'\,\sigma _x\,, \nonumber \\ {\hat{T}}^\zeta _2(u,u')&= u\,\sigma _0 + u'\,\Big (\cos \frac{2\pi }{3}\;\sigma _x +\zeta \,\sin \frac{2\pi }{3}\;\sigma _y\Big )\,, \nonumber \\ {\hat{T}}^\zeta _3(u,u')&= u\,\sigma _0 + u'\,\Big (\cos \frac{2\pi }{3}\;\sigma _x -\zeta \,\sin \frac{2\pi }{3}\;\sigma _y\Big ) = \sigma _x\;{\hat{T}}^\zeta _2(u,u')\;\sigma _x \,. \end{aligned}$$

In particular,

24 $$\begin{aligned} {\hat{T}}^{-\zeta }_i(u,u')&= \sigma _x\,{\hat{T}}^{\zeta }_i(u,u')\,\sigma _x\,. \end{aligned}$$

One can further simplify the notation introducing the Pauli matrices $\tau _a$, $a=0,x,y,z$, with $\tau _0$ the identity, that act in the valley subspace, and thus write

25 $$\begin{aligned} \hat{H}_{{\mathbf {Q}}{\mathbf {Q}}'}({\mathbf {k}})&= \delta _{{\mathbf {Q}}{\mathbf {Q}}'}\,v\,\tau _z\,\big ({\mathbf {k}}-{\mathbf {Q}}\big ) \cdot \,\varOmega \,\varvec{\sigma }\,\varOmega \nonumber \\&\qquad + \tau _0\,\sum _{i=1}^3\,\Big (\delta _{{\mathbf {Q}}'-{\mathbf {Q}},{\mathbf {q}}_i} + \delta _{{\mathbf {Q}}-{\mathbf {Q}}',{\mathbf {q}}_i}\Big )\;\varOmega \,{\hat{T}}_i(u,u')\,\varOmega \,, \end{aligned}$$

where ${\hat{T}}_i(u,u') \equiv {\hat{T}}^{+1}_i(u,u')$, and $\varOmega$ is the real unitary operator

26 $$\begin{aligned} \varOmega&= \sigma _x\;\frac{\displaystyle 1-\tau _z}{\displaystyle 2} + \sigma _0\;\frac{\displaystyle 1+\tau _z}{\displaystyle 2} = \sigma _x\,\text {P}_{\zeta =-1} + \sigma _0\,\text {P}_{\zeta =+1}\;, \end{aligned}$$

being P$_\zeta$ the projector onto valley $\zeta$, which actually interchanges sublattice *A* with *B* in the valley $\zeta =-1$. Applying the unitary operator $\varOmega$, we thus obtain

27 $$\begin{aligned} \varOmega \,\hat{H}_{{\mathbf {Q}}{\mathbf {Q}}'}({\mathbf {k}})\,\varOmega&\rightarrow \hat{H}_{{\mathbf {Q}}{\mathbf {Q}}'}({\mathbf {k}}) = \delta _{{\mathbf {Q}}{\mathbf {Q}}'}\,v\,\tau _z\,\big ({\mathbf {k}}-{\mathbf {Q}}\big )\cdot \,\varvec{\sigma }\, \nonumber \\&\qquad + \tau _0\,\sum _{i=1}^3\,\Big (\delta _{{\mathbf {Q}}'-{\mathbf {Q}},{\mathbf {q}}_i} + \delta _{{\mathbf {Q}}-{\mathbf {Q}}',{\mathbf {q}}_i}\Big )\;{\hat{T}}_i(u,u')\,, \end{aligned}$$

which has the advantage of having a very compact form. For convenience, we list the action of $\varOmega$ applied to $\sigma$ and $\tau$ operators,

28 $$\begin{aligned} \varOmega \,\sigma _x\,\varOmega&= \sigma _x\,, \quad \varOmega \,\sigma _y\,\varOmega = \sigma _y\,\tau _z\,,\quad \varOmega \,\sigma _z\,\varOmega = \sigma _z\,\tau _z\,, \nonumber \\ \varOmega \,\tau _x\,\varOmega&= \tau _x\,\sigma _x\,, \quad \varOmega \,\tau _y\,\varOmega = \tau _y\,\sigma _x\,, \quad \varOmega \,\tau _z\,\varOmega = \tau _z\,. \end{aligned}$$

In this representation, any symmetry operation $\text {G}\in D_6$ corresponds to a transformation

29 $$\begin{aligned} \hat{H}({\mathbf {k}})&= \hat{D}^\dagger \big (\text {G}\big )\, \hat{H} \Big (\text {G}({\mathbf {k}})\Big )\,\hat{D}\big (\text {G}\big )\,, \end{aligned}$$

whose explicit expressions are given in Ref. [32].

## Perturbation induced by a static atomic displacement

We now move to derive in the continuum model the expression of the perturbation induced by a collective atomic displacement. Under a generic lattice deformation, the in-plane atomic positions ${\mathbf {x}}_{i\alpha }$ change according to

30 $$\begin{aligned} {\mathbf {x}}_{i\alpha } \equiv {\mathbf {R}}_i+{\mathbf {r}}_{i\alpha }&\rightarrow {\mathbf {R}}_i+{\mathbf {r}}_\alpha + {\varvec{u}}_i\big ({\mathbf {x}}_{i\alpha }\big ) = {\mathbf {x}}_{i\alpha } + {\varvec{u}}_i\big ({\mathbf {x}}_{i\alpha }\big ) \,, \end{aligned}$$

where *i* is now labelling a generic unit cell position. Since the phonon modes we are going to study involve only in-plane atomic displacements, we assume that *z*-coordinate of each carbon atom does not vary. It follows that a generic potential in the two-centre approximation and at linear order in the displacement reads

31 $$\begin{aligned}&T\Big ({\mathbf {x}}_{i\alpha }-{\mathbf {x}}_{j\beta }, z_{i\alpha }-z_{j\beta } \Big )\rightarrow T\Big ({\mathbf {x}}_{i\alpha }-{\mathbf {x}}_{j\beta }, z_{i\alpha }-z_{j\beta }\Big ) \nonumber \\&\qquad \qquad + {\varvec{W}}\Big ({\mathbf {x}}_{i\alpha }-{\mathbf {x}}_{j\beta }, z_{i\alpha }-z_{j\beta }\Big )\cdot \Big ({\varvec{u}}_i\big ({\mathbf {x}}_{i\alpha }\big ) -{\varvec{u}}_j\big ({\mathbf {x}}_{j\beta }\big )\Big ) \,. \end{aligned}$$

We further neglect the dependence on *z*, which we will take into account by distinguishing at the end between different scattering channels, intra- and inter-layers, so that:

32 $$\begin{aligned} {\varvec{W}}({\mathbf {r}})&= \frac{\displaystyle 1}{\displaystyle N}\,\sum _{\mathbf {q}}\, \text {e}^{i{\mathbf {q}}\cdot {\mathbf {r}}}\;{\varvec{W}}({\mathbf {q}}) = {\varvec{\nabla }}\,T({\mathbf {r}}) = i\;\frac{\displaystyle 1}{\displaystyle N}\,\sum _{\mathbf {q}}\, {\mathbf {q}}\;T({\mathbf {q}})\,\text {e}^{i{\mathbf {q}}\cdot {\mathbf {r}}}\,, \end{aligned}$$

namely

33 $$\begin{aligned} {\varvec{W}}({\mathbf {q}}) = i{\mathbf {q}}\,T({\mathbf {q}}) = i{\mathbf {q}}\,T(q)\,, \end{aligned}$$

assuming, as before, that $T({\mathbf {q}})$ depends only on $q=|{\mathbf {q}}|$.

**Fig. 3:**
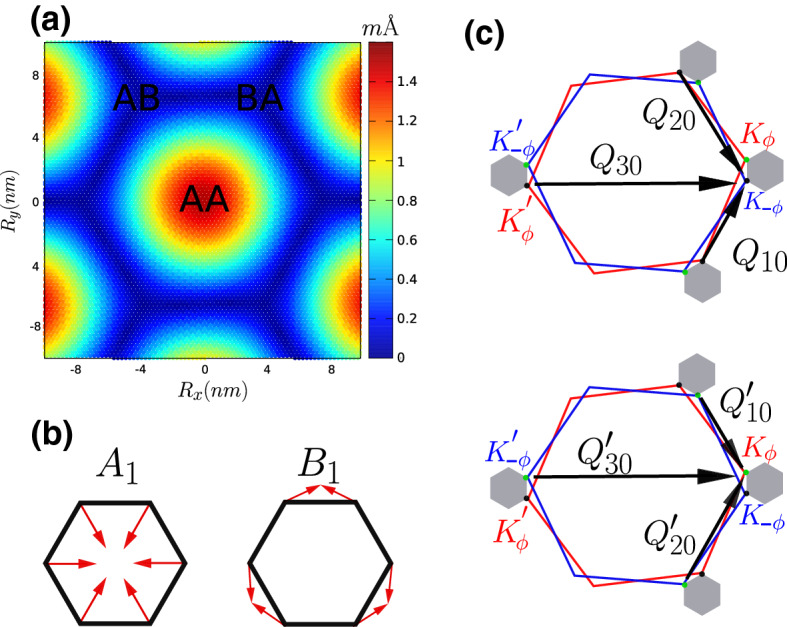
**a** Real space phonon amplitude of the pair of Jahn–Teller phonon modes found in Ref. [8]. These modes, often referred to as “moiré modes”, vibrate mostly within the AA regions involving no atomic movement at all within the Bernal (AB/BA)-stacked regions. **b** Sketch of the microscopic vibration of these modes. The atomic movement is in-plane and behaves as two irreducible representations ($A_1$ and $B_1$) of the group $\text {D}_6$. **c** *Q*-vectors which connect inequivalent valleys in different layers. These vectors has been used to approximate the vibration of the modes in **a** and **b**, see (34)

###  Jahn–Teller moiré phonon modes

Reference [8] pointed out the existence of a pair of high-frequency optical modes at the ${\varvec{\varGamma }}$ point of the MBZ, which are extremely efficient in lifting the valley degeneracies observed in the band structure. These phonon modes are schematically drawn in Fig.3a, and they both share the same modulation on the moiré length scale. However, microscopically, they both look as the well-known in-plane optical phonon modes of graphene at ${\mathbf {K}}$, which transform as the $A_1$ and $B_1$ irreducible representations, see Fig.3b. These two irreducible representations differ by the fact that $B_1$ is odd with respect to $\text {C}_{2z}$ and $\text {C}_{2y}$, while $A_1$ is even with respect to all symmetries of the $\text {D}_6$ space group.

Although the complexity of these modes is hard to capture by a simple analytical expression, their effect on the band structure can be well approximated introducing the following deformation fields

34 $$\begin{aligned} \begin{aligned} {\varvec{u}}_c^{(a)}\Big ({\mathbf {x}}^{(a)}_\alpha \Big )&= \sum _{i=1}^3\,\sum _{j=0}^2\, {\varvec{u}}^{(a)}_{\alpha }\big ({\mathbf {Q}}_{ij} \big )\,\cos \Big (\big ({\mathbf {R}}^{(a)}+{\mathbf {r}}^{(a)}_\alpha \big ) \cdot {\mathbf {Q}}_{ij}\Big )\,, \\ {\varvec{u}}_s^{(a)}\Big ({\mathbf {x}}^{(a)}_\alpha \Big )&= \sum _{i=1}^3\,\sum _{j=0}^2\, {\varvec{v}}^{(a)}_{\alpha } \big ({\mathbf {Q}}_{ij} \big )\,\sin \Big (\big ({\mathbf {R}}^{(a)}+{\mathbf {r}}^{(a)}_\alpha \big ) \cdot {\mathbf {Q}}_{ij}\Big )\,, \end{aligned} \end{aligned}$$

where ${\mathbf {Q}}_{ij}$ are the k-vectors connecting different valleys and depicted in Fig. 3c, while $a=1,2$ is the layer index. Since the transformation $\text {C}_{g}$ ($g=3z,2x$) is a symmetry operation even in the distorted lattice, we have that

35 $$\begin{aligned} \text {C}_{g}\Big ({\mathbf {x}}^{(a)}_\alpha \Big )&= {\mathbf {x}}^{(b)}_\beta \, \longrightarrow \, \text {C}_{g}\Big ({\varvec{u}}^{(a)}\big ({\mathbf {x}}^{(a)}_\alpha \big )\Big ) = {\varvec{u}}^{(b)}\big ({\mathbf {x}}^{(b)}_\beta \big )\,. \end{aligned}$$

By noting that the set of momenta $\{{\mathbf {Q}}_{ij}\}$ is invariant under $\text {C}_{3z}$ and $\text {C}_{2x}$, it immediately follows that, for $g=3z,2x$

36 $$\begin{aligned} \begin{aligned} \text {C}_{g}\Big ({\varvec{u}}^{(a)}_{\alpha }\big ({\mathbf {Q}}_{ij}\big )\Big )&= {\varvec{u}}^{(b)}_{\beta }\Big (\text {C}_{g}\big ({\mathbf {Q}}_{ij}\big )\Big )\,. \\ \text {C}_{g}\Big ({\varvec{v}}^{(a)}_{\alpha }\big ({\mathbf {Q}}_{ij}\big )\Big )&= {\varvec{v}}^{(b)}_{\beta }\Big (\text {C}_{g}\big ({\mathbf {Q}}_{ij}\big )\Big )\,. \end{aligned} \end{aligned}$$

On the contrary, $\{{\mathbf {Q}}_{ij}\}$ is not invariant under $C_g$ ($g=2y,2z$), and the phonon modes are either even ($A_1$) or odd ($B_1$) under these symmetries. Therefore, recalling that $\text {C}_{2z}$ exchanges the two sublattices,

37 $$\begin{aligned} \begin{aligned} \text {C}_{2z}\Big ({\varvec{u}}^{(a)}_{A}\big ({\mathbf {Q}}_{ij}\big )\Big )&= - {\varvec{u}}^{(a)}_{A}\big ({\mathbf {Q}}_{ij}\big ) = \pm {\varvec{u}}^{(a)}_{B}\big ({\mathbf {Q}}_{ij}\big )\,, \\ \text {C}_{2z}\Big ({\varvec{v}}^{(a)}_{A}\big ({\mathbf {Q}}_{ij}\big )\Big )&= - {\varvec{v}}^{(a)}_{A}\big ({\mathbf {Q}}_{ij}\big ) = \mp {\varvec{v}}^{(a)}_{B}\big ({\mathbf {Q}}_{ij}\big )\,. \end{aligned} \end{aligned}$$

If we choose

38 $$\begin{aligned} {\varvec{u}}^{(a)}_{A}\big ({\mathbf {Q}}_{ij}\big )&= {\varvec{u}}^{(a)}_{B}\big ({\mathbf {Q}}_{ij}\big )\,, \quad {\varvec{v}}^{(a)}_{A}\big ({\mathbf {Q}}_{ij}\big ) = {\varvec{v}}^{(a)}_{B}\big ({\mathbf {Q}}_{ij}\big )\,, \end{aligned}$$

then the cosine distortion in (34) ${\varvec{u}}_c^{(a)}\Big ({\mathbf {x}}^{(a)}_\alpha \Big )$ transforms as $B_1$, while the sine one, ${\varvec{u}}_s^{(a)}\Big ({\mathbf {x}}^{(a)}_\alpha \Big )$, as $A_1$. They both can be shortly written as

39 $$\begin{aligned} {\varvec{u}}^{(a)}\Big ({\mathbf {x}}^{(a)}_\alpha \Big )&= \sum _{i=1}^3\,\sum _{j=0}^2\, \Bigg [ {\varvec{u}}^{(a)}\Big ({\mathbf {Q}}_{ij}\Big )\,\text {e}^{i\big ({\mathbf {R}}^{(a)}+{\mathbf {r}}^{(a)}_\alpha \big ) \cdot {\mathbf {Q}}_{ij}}\;+\,c.c.\Bigg ]\,, \end{aligned}$$

where ${\varvec{u}}^{(a)}\big ({\mathbf {Q}}_{ij}\big ){^*} \equiv {\varvec{u}}^{(a)}\big (-{\mathbf {Q}}_{ij}\big )$ and ${\varvec{u}}^{(a)}\big ({\mathbf {Q}}_{ij}\big )$ is real for the $B_1$ distortion and imaginary for $A_1$.

We end by pointing out that ${\varvec{W}}({\mathbf {q}})$ satisfies

40 $$\begin{aligned} \text {C}_{g}\big ({\varvec{W}}({\mathbf {q}})\big )&= {\varvec{W}}\Big (\text {C}_{g}\big ({\mathbf {q}}\big )\Big )\,, \end{aligned}$$

for all symmetry operations of the lattice, in particular

41 $$\begin{aligned} \text {C}_{2z}\Big ({\varvec{W}}({\mathbf {q}})\Big )&= -{\varvec{W}}({\mathbf {q}}) = {\varvec{W}}\Big (\text {C}_{2z}\big ({\mathbf {q}}\big )\Big ) = {\varvec{W}}(-{\mathbf {q}}) = {\varvec{W}}({\mathbf {q}})^{*} \,. \end{aligned}$$

### Phonon-induced Hamiltonian matrix elements

A lattice distortion involving the $A_1$ or $B_1$ phonons generates a matrix element between layer *a* momentum ${\mathbf {k}}\sim {\mathbf {K}}^{(a)}$ and layer *b* momentum ${\mathbf {p}}\sim -{\mathbf {K}}^{(b)}$, where we recall that ${\mathbf {K}}^{(1)}={\mathbf {K}}_2$ and ${\mathbf {K}}^{(2)}={\mathbf {K}}_1$ in Fig. 1d:

42 $$\begin{aligned}&W(a,{\mathbf {k}},\alpha ;b,{\mathbf {p}},\beta ) = \frac{\displaystyle 1}{\displaystyle N^2}\sum _{{\mathbf {q}}{\mathbf {Q}}_{ij}}\sum _{{\mathbf {R}}^{(a)}{\mathbf {R}}^{(b)}} \!\!\!{\varvec{W}}(-{\mathbf {q}})\cdot \Bigg [{\varvec{u}}^{(a)}\Big ({\mathbf {Q}}_{ij}\Big )\, \text {e}^{-i\big ({\mathbf {k}}+{\mathbf {q}}-{\mathbf {Q}}_{ij}\big )\cdot \big ({\mathbf {R}}^{(a)}+{\mathbf {r}}^{(a)}_\alpha \big )}\; \text {e}^{i({\mathbf {p}}+{\mathbf {q}})\cdot \big ({\mathbf {R}}^{(b)}+{\mathbf {r}}^{(b)}_\beta \big )}\nonumber \\&\qquad - {\varvec{u}}^{(b)}\Big ({\mathbf {Q}}_{ij}\Big )\,\text {e}^{-i({\mathbf {k}}+{\mathbf {q}})\cdot \big ({\mathbf {R}}^{(a)}+{\mathbf {r}}^{(a)}_\alpha \big )}\; \text {e}^{i\big ({\mathbf {p}}+{\mathbf {q}}+{\mathbf {Q}}_{ij}\big )\cdot \big ({\mathbf {R}}^{(b)}+{\mathbf {r}}^{(b)}_\beta \big )} \Bigg ] \nonumber \\&\quad = \sum _{{\mathbf {q}}{\mathbf {Q}}_{ij}}\sum _{{\mathbf {G}}^{(a)}{\mathbf {G}}^{(b)}}\!\! {\varvec{W}}(-{\mathbf {q}})\cdot \Bigg [ \delta _{-{\mathbf {q}},{\mathbf {k}}-{\mathbf {Q}}_{ij}+{\mathbf {G}}^{(a)}}\, \delta _{-{\mathbf {q}},{\mathbf {p}}+{\mathbf {G}}^{(b)}}\; {\varvec{u}}^{(a)}\Big ({\mathbf {Q}}_{ij}\Big )\; \text {e}^{i{\mathbf {G}}^{(a)}\cdot {\mathbf {r}}^{(a)}_\alpha -i{\mathbf {G}}^{(b)}\cdot {\mathbf {r}}^{(b)}_\beta } \nonumber \\&\qquad - \delta _{-{\mathbf {q}},{\mathbf {k}}+{\mathbf {G}}^{(a)}}\, \delta _{-{\mathbf {q}},{\mathbf {p}}+{\mathbf {Q}}_{ij}+{\mathbf {G}}^{(b)}}\; {\varvec{u}}^{(b)}\Big ({\mathbf {Q}}_{ij}\Big )\; \text {e}^{i{\mathbf {G}}^{(a)}\cdot {\mathbf {r}}^{(a)}_\alpha -i{\mathbf {G}}^{(b)}\cdot {\mathbf {r}}^{(b)}_\beta }\;\Bigg ]\,. \end{aligned}$$

We can readily follow the same steps outlined in Sect. 2 to identify the ${\mathbf {G}}^{(a)}$ and ${\mathbf {G}}^{(b)}$ reciprocal lattice vectors that enforce momentum conservation and maximise the matrix element $W(-{\mathbf {q}})=W(q)$. Therefore, we shall not repeat that calculation and jump directly to the results.

The lattice distortion introduces a perturbation both intra-layer and inter-layer. The former, in the representation introduced in Sect. 2.1, has the extremely simple expression:

43 $$\begin{aligned} \delta {\hat{H}}_{x(y)}^{||}({\mathbf {k}}){_{{\mathbf {Q}}{\mathbf {Q}}'}}&= \tau _{x(y)}\,\sum _{i=1}^3\,\Big (\delta _{{\mathbf {Q}}'-{\mathbf {Q}},{\mathbf {q}}_i} + \delta _{{\mathbf {Q}}-{\mathbf {Q}}',{\mathbf {q}}_i}\Big )\;{\hat{T}}_i(g,g') \equiv \tau _{x(y)}\;\delta {\hat{H}}^{||}_{{\mathbf {Q}}{\mathbf {Q}}'} \,, \end{aligned}$$

where $\tau _x$ refers to the $A_1$ mode, $\tau _y$ to the $B_1$ one, and the matrices ${\hat{T}}_i(g,g')$ have the same expression as those in Eq. (14), with *u* and $u'$ replaced, respectively, by *g* and $g'$.

The inter-layer coupling has a simpler expression, since, as we many times mentioned, the opposite valleys in different layers fold on the same momentum in the MBZ, and thus, the coupling is diagonal in ${\mathbf {Q}}$ and ${\mathbf {Q}}'$ and reads

44 $$\begin{aligned} \delta {\hat{H}}_{x(y)}^{\perp }({\mathbf {k}})_{{\mathbf {Q}}{\mathbf {Q}}'}&= \delta _{{\mathbf {Q}},{\mathbf {Q}}'}\;\gamma \,\sigma _0\,\tau _{x(y)} \equiv \tau _{x(y)}\;\delta {\hat{H}}^{\perp }_{{\mathbf {Q}}{\mathbf {Q}}'}\,. \end{aligned}$$

As before, $\tau _x$ and $\tau _y$ refers to the $A_1$ and $B_1$ modes, respectively.

It is worth remarking that, because of the transformation (26), which exchanges the sublattices in the valley $\zeta =-1$, the diagonal elements of the matrices ${\hat{T}}_i(g,g')$ in (43) and $\sigma _0$ in (44) refer to the opposite sublattices, while the diagonal elements to the same sublattice, right the opposite of the unperturbed Hamiltonian (27).

Let us rephrase the above results in the second quantisation and introducing the quantum mechanical character of the phonon mode. In the continuum model, a plane wave with momentum ${\mathbf {k}}+{\mathbf {G}}$, where ${\mathbf {G}}=n{\varvec{A}}+m{\varvec{B}}$ is a reciprocal lattice vector of the MBZ, in layer $i=1,2$, valley $\zeta =+1$ and with sublattice components described by a two-component spinor $\chi _{{\mathbf {k}}+{\mathbf {G}}}$, can be associated with a two-component spinor operator according to

45 $$\begin{aligned} \text {e}^{i({\mathbf {k}}+{\mathbf {G}})\cdot {\mathbf {r}}}\;\chi _{{\mathbf {k}}+{\mathbf {G}}}\; \xrightarrow \;\; \varPsi ^{(i)}_{+1,{\mathbf {k}}+{\mathbf {G}}}\,. \end{aligned}$$

For any ${\mathbf {G}}$, we can write, see Eq. (17),

46 $$\begin{aligned} {\mathbf {G}}&= {\mathbf {K}}_1 -{\mathbf {Q}}_A = {\mathbf {K}}_2-{\mathbf {Q}}_B\,, \end{aligned}$$

and thus define

47 $$\begin{aligned} \varPsi ^{(1)}_{+1,{\mathbf {k}}+{\mathbf {G}}}&= \varPsi ^{(1)}_{+1,{\mathbf {k}}+{\mathbf {K}}_{2}-{\mathbf {Q}}_B} \equiv \varPsi _{{\mathbf {k}},{\mathbf {Q}}_B,+1}\,, \nonumber \\ \varPsi ^{(2)}_{+1,{\mathbf {k}}+{\mathbf {G}}}&= \varPsi ^{(1)}_{+1,{\mathbf {k}}+{\mathbf {K}}_{1}-{\mathbf {Q}}_A} \equiv \varPsi _{{\mathbf {k}},{\mathbf {Q}}_A,+1}\,. \end{aligned}$$

We note that ${\mathbf {K}}_{+\phi }+{\mathbf {K}}_{-\phi }\equiv {\mathbf {G}}_k = (2k+1)\,\big ({\varvec{A}}+{\varvec{B}}\big )$, which allows us defining the operators in valley $\zeta =-1$ as

48 $$\begin{aligned} \varPsi ^{(1)}_{-1,{\mathbf {k}}+{\mathbf {G}}-{\mathbf {G}}_k}&= \varPsi ^{(1)}_{-1,{\mathbf {k}}-{\mathbf {K}}_{+\phi }-{\mathbf {Q}}_A} \equiv \sigma _x\,\varPsi _{{\mathbf {k}},{\mathbf {Q}}_A,-1}\,, \nonumber \\ \varPsi ^{(2)}_{-1,{\mathbf {k}}+{\mathbf {G}}-{\mathbf {G}}_k}&= \varPsi ^{(2)}_{+1,{\mathbf {k}}-{\mathbf {K}}_{-\phi }-{\mathbf {Q}}_B} \equiv \sigma _x\,\varPsi _{{\mathbf {k}},{\mathbf {Q}}_B,-1}\,, \end{aligned}$$

where, in accordance with our transformation in Eq. (26), we interchange the two sublattices in valley $\zeta =-1$ through $\sigma _x$. We note that the mismatch momentum ${\mathbf {G}}_k$ is just what is provided by the phonon modes. Absorbing the valley index into two additional components of the spinors, and introducing back the spin label, the second quantised Hamiltonian can be written in terms of four-component spinor operators $\varPsi _{{\mathbf {k}}\sigma ,{\mathbf {Q}}}$, where, ${\mathbf {Q}}={\mathbf {Q}}_A$ refer to layer 2 if the valley index $\zeta =+1$ and layer 1 if $\zeta =-1$, while ${\mathbf {Q}}={\mathbf {Q}}_B$ to layer 1 if $\zeta =+1$, and layer 2 if $\zeta =-1$.

Next, we introduce a two-component dimensionless variable ${\mathbf {q}}_{\varvec{0}}=(q_1, q_2)$, and its conjugate one, ${\mathbf {p}}_{\varvec{0}}=(p_1, p_2)$, where $q_1$ and $q_2$ are the phonon coordinates of the $A_1$ and $B_1$ modes at ${\varvec{\varGamma }}={\varvec{0}}$, respectively. Using the above-defined operators, the full quantum mechanical Hamiltonian reads

49 $$\begin{aligned} H&= \sum _{{\mathbf {k}}{\mathbf {Q}}\sigma }\, \bigg [\;\varPsi ^\dagger _{{\mathbf {k}}\sigma \,{\mathbf {Q}}}\;{\hat{H}}_{{\mathbf {Q}}{\mathbf {Q}}'}({\mathbf {k}})\; \varPsi _{{\mathbf {k}}\sigma \,{\mathbf {Q}}'} + \varPsi ^\dagger _{{\mathbf {k}}\sigma \,{\mathbf {Q}}} \Big ( {\varvec{q}}_{\varvec{0}}\cdot {\varvec{\tau }}\;\delta {\hat{H}}_{{\mathbf {Q}}{\mathbf {Q}}'} \Big ) \,\varPsi _{{\mathbf {k}}\sigma \,{\mathbf {Q}}'}\;\bigg ] \nonumber \\&\qquad \qquad \qquad + \frac{\displaystyle \omega _{\varvec{0}}}{\displaystyle 2}\,\Big (\,{\mathbf {p}}_{\varvec{0}}\cdot {\mathbf {p}}_{\varvec{0}} + {\mathbf {q}}_{\varvec{0}}\cdot {\mathbf {q}}_{\varvec{0}}\Big )\,, \end{aligned}$$

where $\omega _{\varvec{0}}$ is the phonon frequency, equal for both $A_1$ and $B_1$ modes, ${\varvec{\tau }}=(\tau _x,\tau _y)$, and

50 $$\begin{aligned} \hat{H}_{{\mathbf {Q}}{\mathbf {Q}}'}({\mathbf {k}})&= \delta _{{\mathbf {Q}}{\mathbf {Q}}'}\,v\,\tau _z\,\big ({\mathbf {k}}-{\mathbf {Q}}\big )\cdot \,\varvec{\sigma }+ \tau _0\,\sum _{i=1}^3\,\Big (\delta _{{\mathbf {Q}}'-{\mathbf {Q}},{\mathbf {q}}_i} + \delta _{{\mathbf {Q}}-{\mathbf {Q}}',{\mathbf {q}}_i}\Big )\;{\hat{T}}_i(u,u')\,, \nonumber \\ \delta {\hat{H}}_{{\mathbf {Q}}{\mathbf {Q}}'}&= \delta {\hat{H}}^{||}_{{\mathbf {Q}}{\mathbf {Q}}'} + \delta {\hat{H}}^{\perp }_{{\mathbf {Q}}{\mathbf {Q}}'} = \sum _{i=1}^3\,\Big (\delta _{{\mathbf {Q}}'-{\mathbf {Q}},{\mathbf {q}}_i} + \delta _{{\mathbf {Q}}-{\mathbf {Q}}',{\mathbf {q}}_i}\Big )\;{\hat{T}}_i(g,g') + \delta _{{\mathbf {Q}},{\mathbf {Q}}'}\;\gamma \,. \end{aligned}$$

We observe that the Hamiltonian (49) still possesses a valley $U_v(1)$ symmetry, with generator

51 $$\begin{aligned} J_z&= \frac{\displaystyle 1}{\displaystyle 2}\,\sum _{{\mathbf {k}}{\mathbf {Q}}\sigma }\,\varPsi ^\dagger _{{\mathbf {k}}\sigma \,{\mathbf {Q}}}\;\sigma _0\,\tau _z \;\varPsi _{{\mathbf {k}}\sigma \,{\mathbf {Q}}} + {\mathbf {q}}_{\varvec{0}}\wedge {\mathbf {p}}_{\varvec{0}} \equiv T_z + L_z\,, \end{aligned}$$

where $T_z$ is half the difference between the number of electrons in valley $\zeta =+1$ and the one in valley $\zeta =-1$, while $L_z$ is the angular momentum of the phonon mode. The Hamiltonian (49) actually realises a $e\otimes E$ Jahn–Teller model.

It is straightforward to generalise the above result to an atomic displacement modulated with the wave vectors ${\mathbf {Q}}_{ij} + {\varvec{P}}$, where ${\varvec{P}}\in \text {MBZ}$. Since ${\mathbf {Q}}_{ij}$ are multiples of the MBZ reciprocal lattice vector, such displacement is at momentum ${\varvec{P}}$ and can be considered as the previous one at ${\varvec{\varGamma }}$, as shown in Fig. 3, on top of which we add an additional incommensurate long wavelength component. Since ${\varvec{P}}$ is tiny as compared to the vectors ${\mathbf {Q}}_{ij}$, we shall assume that the displacement has the same expression of Eq. (39), with the only difference that

52 $$\begin{aligned} \text {e}^{\pm i\big ({\mathbf {R}}^{(a)}+{\mathbf {r}}^{(a)}_\alpha \big ) \cdot {\mathbf {Q}}_{ij}} \;&\xrightarrow \,\; \text {e}^{\pm i\big ({\mathbf {R}}^{(a)}+{\mathbf {r}}^{(a)}_\alpha \big ) \cdot \big ({\mathbf {Q}}_{ij}\pm {\varvec{P}}\big )} \;. \end{aligned}$$

The full quantum mechanical Hamiltonian becomes

53 $$\begin{aligned} H&= \sum _{{\mathbf {k}}{\mathbf {Q}}\sigma }\, \varPsi ^\dagger _{{\mathbf {k}}\sigma \,{\mathbf {Q}}}\;{\hat{H}}_{{\mathbf {Q}}{\mathbf {Q}}'}({\mathbf {k}})\; \varPsi _{{\mathbf {k}}\sigma \,{\mathbf {Q}}'} + \sum _{{\mathbf {k}}{\mathbf {Q}}{\varvec{P}}\sigma }\, \varPsi ^\dagger _{{\mathbf {k}}\sigma \,{\mathbf {Q}}} \Big ( {\varvec{q}}_{-{\varvec{P}}}\cdot {\varvec{\tau }}\;\delta {\hat{H}}_{{\mathbf {Q}}{\mathbf {Q}}'}({\varvec{P}}) \Big ) \,\varPsi _{{\mathbf {k}}+{\varvec{P}}\sigma \,{\mathbf {Q}}'} \nonumber \\&\quad + \frac{\displaystyle 1}{\displaystyle 2}\,\sum _{{\varvec{P}}}\, \omega _{\varvec{P}}\,\Big (\,{\mathbf {p}}_{\varvec{P}}\cdot {\mathbf {p}}_{-{\varvec{P}}} + {\mathbf {q}}_{\varvec{P}}\cdot {\mathbf {q}}_{-{\varvec{P}}}\Big )\,, \end{aligned}$$

where $\delta {\hat{H}}_{{\mathbf {Q}}{\mathbf {Q}}'}({\varvec{P}})$ is the same as $\delta {\hat{H}}_{{\mathbf {Q}}{\mathbf {Q}}'}$ in Eq. (50) with ${\varvec{P}}$-dependent constants $g_{\varvec{P}}$, $g'_{\varvec{P}}$ and $\gamma _{\varvec{P}}$, invariant under the little group at ${\varvec{P}}$. In this general case, the generator of $U_v(1)$ reads

54 $$\begin{aligned} J_z&= \frac{\displaystyle 1}{\displaystyle 2}\,\sum _{{\mathbf {k}}{\mathbf {Q}}\sigma }\,\varPsi ^\dagger _{{\mathbf {k}}\sigma \,{\mathbf {Q}}}\;\sigma _0\,\tau _z \;\varPsi _{{\mathbf {k}}\sigma \,{\mathbf {Q}}} + \sum _{{\varvec{P}}}\,{\mathbf {q}}_{\varvec{P}}\wedge {\mathbf {p}}_{-{\varvec{P}}} \,. \end{aligned}$$

### Frozen phonon band structure

**Fig. 4:**
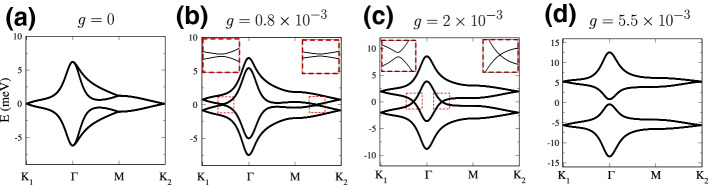
Band structure of $2\phi =1.08^\circ$ twisted bilayer graphene with increasing frozen phonon deformation intensity *g*. The other parameters used in this calculation are: $u=0.0761$, $u'=0.1031$, $g'=g/10$ and $\gamma =g/2.5$. **a** Undistorted case. **b** Slightly distorted lattice. Two small avoided crossings occur along the ${\mathbf {K}}_1 \rightarrow {\varvec{\varGamma }}$ and ${\mathbf {M}}\rightarrow {\varvec{\varGamma }}$ lines. **c** By further increasing the distortion intensity, the four bands further separate. The avoided crossing along ${\mathbf {K}}_1 \rightarrow {\varvec{\varGamma }}$ persists, while a genuine crossing occurs along ${\varvec{\varGamma }}\rightarrow {\mathbf {M}}$. **d** A gap at charge neutrality has finally opened

We can perform a frozen phonon calculation neglecting the phonon energy, last term in Eq. (49), and fixing ${\mathbf {q}}=(q_1,q_2)$ to some constant value. Because of the $U_v(1)$ symmetry, what matters is just the modulus *q* of ${\mathbf {q}}$. In practice, we have taken ${\mathbf {q}}=(1,0)$ and studied the band structure varying the coupling constants *g*, setting $g'=g/10$ and $\gamma =g/2.5$, and assuming the following parameters: $\hbar v/a_0=2.1354\;eV$ [29]; $u=0.0761\; eV$ and $u'=0.1031 \;eV$ [14]. This choice fits well the microscopic tight-binding calculations in Ref. [8]. As shown in Fig. 4, as soon as the frozen phonon terms are turned on, all the degeneracies in the band structure arising due to the valley symmetry are lifted. This occurs with a set of avoided crossings which move from ${\mathbf {K}}\rightarrow {\mathbf {M}}$ and from ${\mathbf {K}}\rightarrow {\varvec{\varGamma }}$ (Fig. 4b). In particular, the crossings that move from ${\mathbf {K}}\rightarrow {\mathbf {M}}$ eventually meet at ${\mathbf {M}}$, forming (six) Dirac nodes, which then move towards ${\varvec{\varGamma }}$ (Fig. 4c). Finally, at a threshold value of *g*, a gap opens at the charge neutrality point (Fig. 4d). Such gap keeps increasing as the deformation amplitude increases.

### Moiré phonons at ${\mathbf {M}}$

The phonon modes considered in the previous section were at position ${\varvec{\varGamma }}$ of the MBZ, thus preserving the periodicity of the moiré superlattice. As pointed out in Ref. [8] and shown before, these modes are able to open a gap in the band structure only at charge neutrality. Gap opening at different commensurate fillings requires freezing finite momentum phonons [8]. Here, we consider a multicomponent distortion which involves the modes at the three inequivalent ${\mathbf {M}}$ points in the MBZ:

55 $$\begin{aligned} {\mathbf {M}}_1 = \frac{\displaystyle {\varvec{A}}}{\displaystyle 2}\;, \quad {\mathbf {M}}_2 = \text {C}_{3z}\big ({\mathbf {M}}_1 \big )= \frac{\displaystyle {\varvec{B}}}{\displaystyle 2}\;, \quad {\mathbf {M}}_3 = \text {C}_{3z} \big ({\mathbf {M}}_2 \big ) = -\frac{\displaystyle {\varvec{A}}+{\varvec{B}}}{\displaystyle 2}\;. \end{aligned}$$

**Fig. 5:**
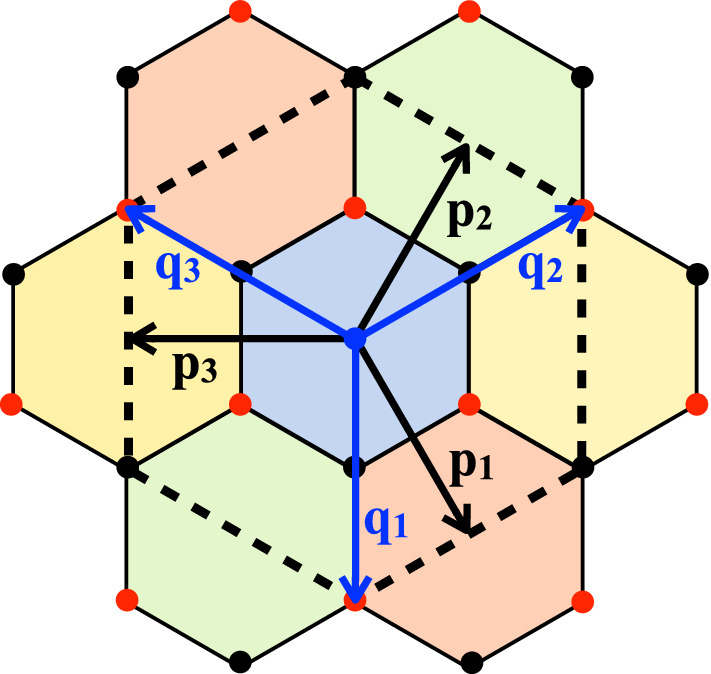
Reduced Brillouin zones with the multicomponent distortion at ${\mathbf {M}}$, shown as coloured hexagons, while the four times larger hexagon drawn with a dashed line is the undistorted MBZ. The vectors ${\mathbf {p}}_i$, black arrows, and ${\mathbf {q}}_i$, blue arrow, for $i=1,\dots ,3$, are also shown. Note that ${\mathbf {p}}_1={\varvec{A}}'$ and ${\mathbf {p}}_2={\varvec{B}}'$ are also the reciprocal lattice vectors of the reduced Brillouin zone. The black and red circles are the positions of the sublattice vectors ${\mathbf {Q}}_A$ and ${\mathbf {Q}}_B$, respectively, defined in Eq. (61)

Freezing a multiple distortion at all these points reduces by a quarter the Brillouin zone, see Fig. 5, which has now the reciprocal lattice vectors

56 $$\begin{aligned} {\varvec{A}}' = {\mathbf {M}}_1 \;,\quad {\varvec{B}}' = {\mathbf {M}}_2\;. \end{aligned}$$

Since ${\mathbf {M}}_i$, $i=1,2,3$, are tiny as compared to the vectors ${\mathbf {Q}}_{ij}$ introduced in the previous section, we can make the same assumption (52) that leads to the Hamiltonian (53); namely, assume that the displacement induced by the multiple distortion has the same expression of Eq. (39), with the only difference that

57 $$\begin{aligned} \text {e}^{i\big ({\mathbf {R}}^{(a)}+{\mathbf {r}}^{(a)}_\alpha \big ) \cdot {\mathbf {Q}}_{ij}} \; \longrightarrow \,\; \sum _{n=1}^6\,\text {e}^{i\big ({\mathbf {R}}^{(a)}+{\mathbf {r}}^{(a)}_\alpha \big ) \cdot \big ({\mathbf {Q}}_{ij}+{\mathbf {p}}_n\big )} \end{aligned}$$

where

58 $$\begin{aligned} {\mathbf {p}}_1 =-{\mathbf {p}}_4={\varvec{M}}_1={\varvec{A}}'\,, \quad {\mathbf {p}}_2 = -{\mathbf {p}}_5 = {\varvec{M}}_2={\varvec{B}}'\,,\quad {\mathbf {p}}_3 =-{\mathbf {p}}_6={\varvec{M}}_3=-{\varvec{A}}'-{\varvec{B}}'\, \end{aligned}$$

are the additional long wavelength modulation vectors on top of the leading short wavelength ones at ${\mathbf {Q}}_{ij}$. The vectors ${\mathbf {q}}_i$ defined in Eq. (18) can be written in terms of the new reciprocal lattice vectors ${\varvec{A}}'$ and ${\varvec{B}}'$ as

59 $$\begin{aligned} {\mathbf {q}}_1 = \frac{\displaystyle 2}{\displaystyle 3}\;\big ({\varvec{A}}'-{\varvec{B}}'\big ) \,, \quad {\mathbf {q}}_2 = \frac{\displaystyle 2}{\displaystyle 3}\;\big ({\varvec{A}}'+2{\varvec{B}}'\big )\,, \quad {\mathbf {q}}_3 = \frac{\displaystyle 2}{\displaystyle 3}\;\big (-2{\varvec{A}}'-{\varvec{B}}'\big ) \,. \end{aligned}$$

**Fig. 6:**
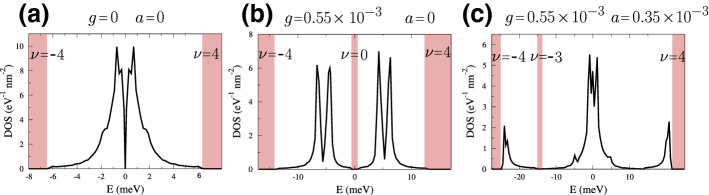
Density of states around charge neutrality obtained with the Hamiltonian (60) with $g'=g/10$, $\gamma =g/2.5$, $a'=a/10$ and $\alpha =a/2.5$. Gaps are highlighted in red, and the corresponding filling factor is $\nu$. a) The undistorted lattice density of states. b) The density of states obtained by deforming the lattice with only the distortion at ${\varvec{\varGamma }}$, which opens a gap at charge neutrality. c) Density of states obtained using both the $\varGamma$ and the ${\mathbf {M}}$ multicomponent distortions. Here, a gap opens at filling of one electron (three holes with respect to neutrality) per unit cell

Both ${\mathbf {p}}_i$ and ${\mathbf {q}}_i$, $i=1,2,3$, are shown in Fig. 5. Considering all momenta ${\mathbf {k}}$ within the new BZ, the light blue hexagon in Fig. 5, and assuming that, besides the multicomponent distortion at ${\mathbf {M}}$, there is still a distortion at ${\varvec{\varGamma }}$, the Hamiltonian can be written again as a matrix $\hat{H}_{{\mathbf {Q}}{\mathbf {Q}}'}({\mathbf {k}})$, which now reads

60 $$\begin{aligned} \hat{H}_{{\mathbf {Q}}{\mathbf {Q}}'}({\mathbf {k}})= & {} \delta _{{\mathbf {Q}},{\mathbf {Q}}'}\; v\,\tau _z\,\Big ({\mathbf {k}}-{\mathbf {Q}}\Big )\cdot \varvec{\sigma }+ \tau _0\,\sum _{i=1}^3\, \Big ( \delta _{{\mathbf {Q}}'-{\mathbf {Q}},{\mathbf {q}}_i} + \delta _{{\mathbf {Q}}-{\mathbf {Q}}',{\mathbf {q}}_i}\Big )\;\hat{T}_i\big (u,u')\nonumber \\&+ \gamma \,\delta _{{\mathbf {Q}},{\mathbf {Q}}'}\;\tau _x + \tau _x\,\sum _{i=1}^3\, \Big ( \delta _{{\mathbf {Q}}'-{\mathbf {Q}},{\mathbf {q}}_i} + \delta _{{\mathbf {Q}}-{\mathbf {Q}}',{\mathbf {q}}_i}\Big )\;\hat{T}_i\big (g,g') \nonumber \\&+\alpha \,\tau _x\sum _{i=1}^6 \Big ( \delta _{{\mathbf {Q}}'-{\mathbf {Q}},{\mathbf {p}}_i} + \delta _{{\mathbf {Q}}-{\mathbf {Q}}',{\mathbf {p}}_i}\Big ) + \tau _x\,\sum _{i=1}^3 \sum _{j=1}^6\Big ( \delta _{{\mathbf {Q}}'-{\mathbf {Q}},{\mathbf {q}}_i+{\mathbf {p}}_j} \nonumber \\&+ \delta _{{\mathbf {Q}}-{\mathbf {Q}}',{\mathbf {q}}_i+{\mathbf {p}}_j}\Big )\;\hat{T}_i\big (a,a')\,, \end{aligned}$$

where the matrices ${\hat{T}}_i(x,x')$ are those in Eq. (14), though they depend on different sets of parameters, $(u,u')$, $(g,g')$ and $(a,a')$. The crucial difference with respect to the Hamiltonian (50) with only the ${\varvec{\varGamma }}$-distortion is that the ${\mathbf {Q}}$ vectors span now the sites of the honeycomb lattice generated by the new fourfold-smaller Brillouin zone; hence, they are defined through

61 $$\begin{aligned} {\mathbf {Q}}&= \Big \{{\mathbf {Q}}'_A\,,\,{\mathbf {Q}}'_B\Big \} = {\left\{ \begin{array}{ll} {\mathbf {Q}}'_A = \frac{\displaystyle {\varvec{A}}'-{\varvec{B}}'}{\displaystyle 3} + n{\varvec{A}}'+m{\varvec{B}}'\,,\\ {\mathbf {Q}}'_B = -\frac{\displaystyle {\varvec{A}}'-{\varvec{B}}'}{\displaystyle 3} + n{\varvec{A}}'+m{\varvec{B}}'\,, \end{array}\right. } \end{aligned}$$

and shown in Fig. 5 as black and red circles, respectively, and must not be confused with those in Eq. (17). In Fig. 6, we show the density of states around neutrality of the Hamiltonian (60). The first two cases correspond to undistorted and $\varGamma$-only distorted structures, while the third panel involves also the *M* multicomponent distortion. As can be seen, a gap now opens at the partial filling of one electron per unit cell. As it was shown in Ref. [8], other phonons or combinations of them can open gaps at any integer filling of the four electronic flat bands.

## Conclusions

We have shown that the moiré phonons of Ref. [8], which are coupled to the valley degrees of freedom of the electrons so to realise an $E\otimes e$ Jahn–Teller model, can be successfully implemented in the continuum model formalism of small-angle twisted bilayer graphene. This method is more manageable than the realistic tight-binding modelling of Ref. [8], whose results have been here reproduced with much less effort. In addition, the continuum model formalism has the great advantage of providing a full quantum mechanical expression of the electron–phonon Hamiltonian, which may allow going beyond the simple frozen phonon calculation of [8], and thus describing phenomena like a dynamical Jahn–Teller effect and the phonon-mediated superconductivity.
